# Supplementary material for: The pre-mRNA splicing modulator pladienolide B inhibits Cryptococcus neoformans germination and growth
Source: mSphere. 2025 Jun 23;10(7):e00248-25. doi: 10.1128/msphere.00248-25 (PMC12306170; doi:10.1128/msphere.00248-25)
Supplement: Supplemental Figures — Figures S1-S3. [file msphere.00248-25-s0001.pdf]

Supplemental Information for
The pre-mRNA Splicing Modulator Pladienolide B Inhibits *Cryptococcus neoformans* Germination and Growth

Sierra L. Love[1,2], Megan C. McKeon[1,3], Henrik Vollmer[2], Joshua C. Paulson[2], Nanami Oshimura[3], Olivia Valentine[3], Sébastien C. Ortiz[3*], Christina M. Hull[3,4], and Aaron A. Hoskins[2,5]

[1]Genetics Training Program, University of Wisconsin-Madison, Madison, WI 53706

[2]Department of Biochemistry, University of Wisconsin-Madison, Madison, WI 53706

[3]Department of Biomolecular Chemistry, University of Wisconsin-Madison, Madison, WI 53706

[4]Department of Medical Microbiology and Immunology, University of Wisconsin-Madison, Madison, WI 53706

[5]Department of Chemistry, University of Wisconsin-Madison, Madison, WI 53706

To whom correspondence should be addressed. Tel: +1 608 890 3101;
Email: ahoskins@wisc.edu

***Current Address:**

Johns Hopkins University

Baltimore, MD

This supplementary file contains:
Supplementary Figures S1-S3 and legends

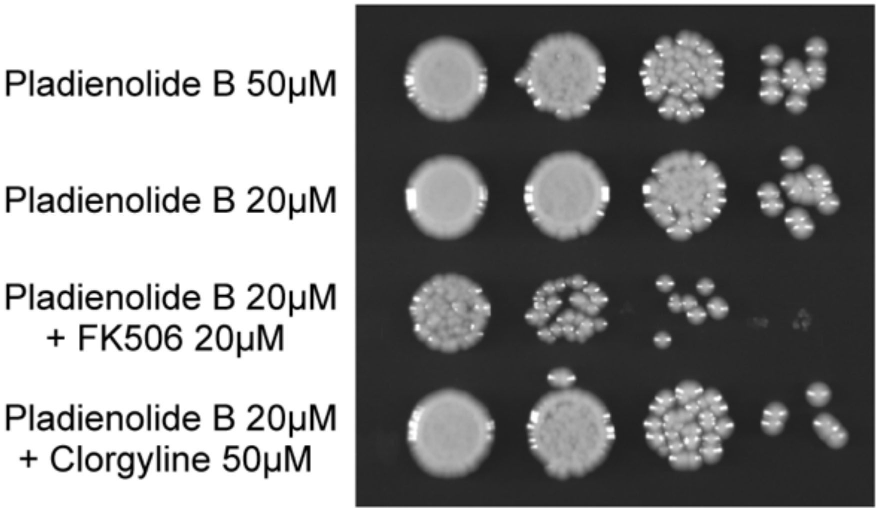

Figure S1. PladB is likely fungistatic, not fungicidal. Stamping assay of serial dilutions of *C. neoformans* strain KN99**α** on solid YPD media after 24 h of exposure to PladB or combinations of PladB with FK506 or clorgyline. Cells were allowed to grow for 3 d after stamping. Colony formation suggests that these drug treatments do not result in the death of all cells within the culture despite growth inhibition.

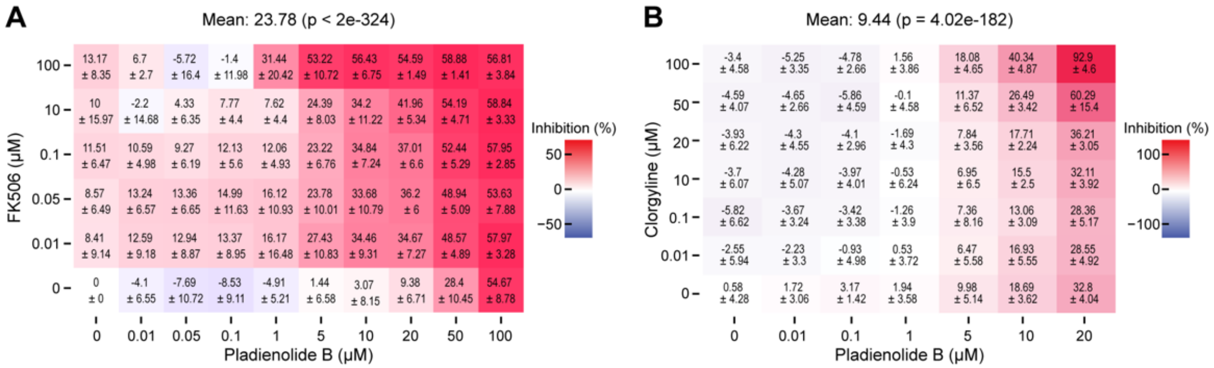

Figure S2. Heatmaps corresponding to data shown in Figures 2F, G. Heatmaps showing percent inhibition of growth of *C. neoformans* strain KN99**α** in combinations of PladB with FK506 (A) or clorgyline (B) relative to DMSO controls. Positive values represent growth inhibition relative to the control, while negative values represent increased growth. Each value represents the average ±SD from $N = 3$ replicates. These values were used to calculate the corresponding synergy scores and contour plots shown in Figure 2.

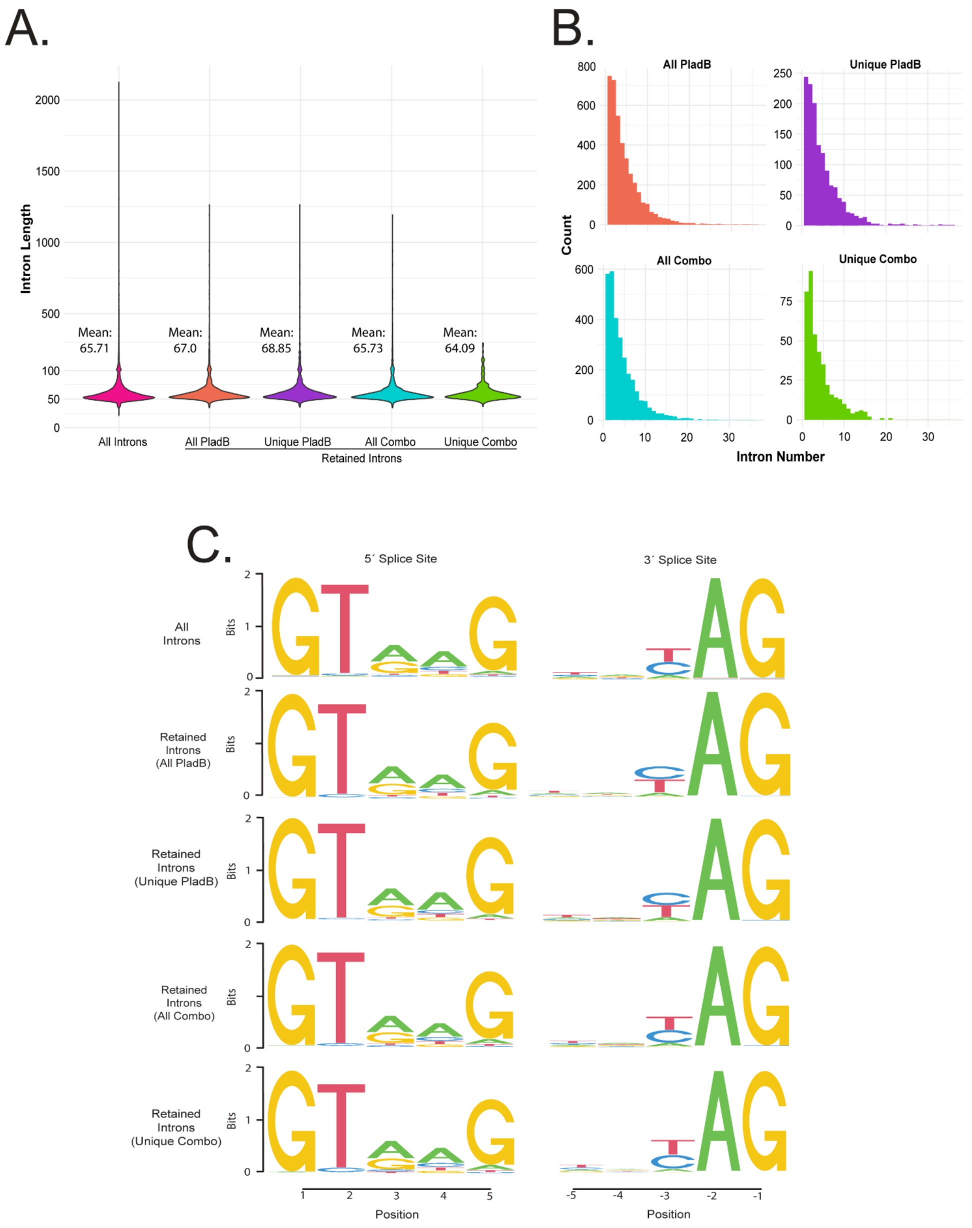

Figure S3. Characteristics of retained introns observed upon drug treatment. (A) Violin plots displaying the distribution of intron lengths for retained introns in the PladB treatment, the PladB + FK506 combination, or those uniquely retained in each condition, relative to all annotated introns in *C. neoformans*. Mean intron lengths for each condition are noted. (B) Histograms depicting the positional distribution of retained introns within genes for each specified condition. (C) Sequence logos of 5′ and 3′ splice sites for all introns and retained introns from each condition listed.
